# Vesicle Nucleation Peptide Fusion Induced Extracellular Vesicles Are Distinct From *Escherichia coli* Outer Membrane Vesicles, and Provide an Enhanced Platform for Protein Production and Purification

**DOI:** 10.1002/jev2.70354

**Published:** 2026-08-14

**Authors:** Bree R. Streather, Tara A. Eastwood, Karen Baker, Mingzhi Liang, Alexandra E. Bailie, Tijn T. van der Velden, Lars J. C. Jeuken, Stanley W. Botchway, Lin Wang, Daniel P. Mulvihill

**Affiliations:** ^1^ School of Biosciences University of Kent Canterbury Kent UK; ^2^ Central Laser Facility, Research Complex at Harwell Science and Technology Facilities Council, Rutherford Appleton Laboratory Didcot Oxford UK; ^3^ Leiden Institute of Chemistry University of Leiden Leiden the Netherlands

**Keywords:** biotechnology, downstream processing, extracellular vesicles, outer membrane vesicles, recombinant protein production

## Abstract

Bacterial outer membrane vesicles (OMVs) are nano‐sized, spherical structures released by Gram‐negative bacteria that play diverse roles in bacterial physiology, including communication, nutrient acquisition and host interactions. These vesicles bud from the bacterial outer membrane and contain lipopolysaccharides, periplasmic proteins, nucleotides and other biomolecules. The vesicle nucleating peptide (VNp) is a short peptide tag that, when fused to the amino terminus of a protein of interest, promotes the formation of bespoke recombinant extracellular vesicles (EVs) in *Escherichia coli*, enabling efficient production and simplified purification of recombinant proteins. Here, we characterise VNp‐induced extracellular vesicles (VNp‐EVs) and compare their composition and organisation with OMVs produced from *E. coli* expressing a periplasmic targeting fusion. While both vesicle types possess a single outer membrane‐derived lipid bilayer, recombinant protein is highly enriched within the VNp‐EVs compared to OMVs containing the periplasm targeting ssDsbA‐fusion protein. VNp‐fusions and the periplasm‐targeted recombinant protein localise to distinct vesicle populations, with VNp‐fusions showing markedly higher luminal concentrations and relative vesicular abundance, compared to the vesicles containing a periplasmic targeted fusion protein. OmpX co‐expression further enriched the VNp‐fusion content of vesicles, further enhancing yield. The VNp‐vesicle lumen is an oxidising environment, thus supports formation of inter‐ and intra‐molecular disulfide bonds within encapsulated proteins. Overall, VNp‐EVs represent a distinct class of recombinant EVs that offer a simple and efficient route for producing and purifying concentrated, correctly folded recombinant proteins, expanding the utility of bacterial vesicle systems for biotechnological applications.

## SIGNIFICANCE STATEMENT

Bacterial extracellular vesicles (EVs) are recognised as versatile tools for biotechnology, yet engineering bacterial vesicle production in a controlled and efficient manner remains challenging. Here we describe how a short vesicle nucleating peptide (VNp) tag, fused to a protein of interest, that can be used to program *Escherichia coli* to produce recombinant EVs that are compositionally and structurally distinct from bacterial outer membrane vesicles (OMVs) containing a periplasm targeted fluorescent protein fusion. VNp‐induced vesicles (VNp‐EVs) are more homogeneous, and more highly enriched in target fusion proteins, providing a simple and efficient route for protein production and purification. The oxidising lumen of these vesicles supports disulfide bond formation, and rapid compartmentalisation enables expression of otherwise challenging or toxic proteins. This work characterises a distinct class of recombinant bacterial vesicles and establishes a practical platform for producing correctly folded, concentrated, partially purified proteins in a self‐packaged form, expanding the potential applications of bacterial EVs in biotechnology and synthetic biology.

## Graphical abstract

In this study, Streather et al. show that the Vesicle Nucleating Peptide (VNp) drives formation of recombinant extracellular vesicles in *E. coli* that are compositionally distinct from native outer membrane vesicles (OMVs). Whereas OMVs contain heterogeneous periplasmic components and low, variable levels of recombinant cargo, Using a range of biophysical analyses they show that VNp‐derived vesicles are densely enriched with target VNp‐fusion proteins. These findings establish VNp as an engineerable platform for producing cargo‐rich recombinant vesicles with potential applications across biotechnology and therapeutic development.

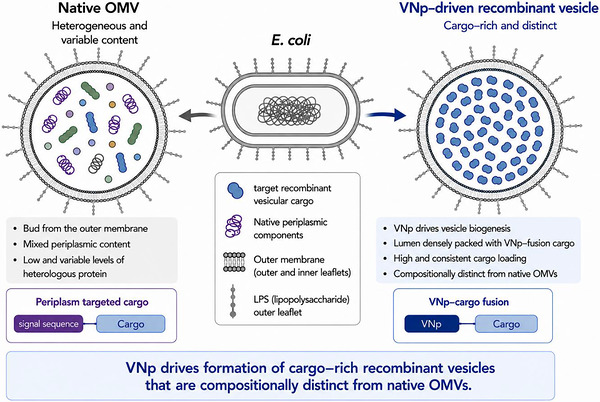

## 1 Introduction

All Gram‐negative bacteria are capable of releasing cellular material into spherical membrane‐bound structures known as outer membrane vesicles (OMVs) (Schwechheimer and Kuehn 2015). They form when a region of the bacterial outer membrane (OM) swells outwards, forming a protuberance that pinches off, without a loss of membrane integrity, and are subsequently released into the extracellular medium. Since their discovery in 1965 (Bishop and Work 1965), various functions have been hypothesised, including cell‐cell communication; virulence factor delivery to host cells; horizontal gene transfer and nutrient breakdown. OMV production is actively regulated by the bacteria in response to cellular stress, nutrient status and interaction with host environments. As well as OM lipids and associated lipopolysaccharides (LPS), OMVs also contain periplasmic proteins, nucleic acids, signalling toxins and, rather than simply being filled with indiscriminate periplasmic content, are enriched for certain proteins (Loeb and Kilner 1979).

It is evident that OMVs play a key role in pathogenicity, primarily through the targeted release of virulence factors, delivered to host cells by endocytosis (Horstman and Kuehn 2000; Kesty and Kuehn 2004). Furthermore, with a membrane composition similar to that of the OM, they can help bacteria to evade the host immune system by acting as a decoy to immune cells. The main immuno‐stimulatory component of the OM is LPS, which makes up the majority of the outer leaflet, making it highly accessible for detection. This molecule consists of a lipid A core, the outer core non‐repeating oligosaccharide and the O‐antigen, another polysaccharide (Raetz and Whitfield 2002). The structure of lipid A and the O‐antigen varies between bacterial strains and species but the core oligosaccharide tends to be well conserved within a genus or family, showing its importance in membrane integrity. The lipid A moiety can be detected at picomolar concentrations by the toll‐like‐receptor 4 (TLR4) in the host innate immune system causing the release of inflammatory cytokines and activation of many downstream processes, potentially leading to endotoxic shock, which can be fatal. The O‐antigen triggers the complement cascade, but a longer O‐antigen can confer resistance to the effects of this (Osawa et al. 2013). LPS has been identified in OMVs produced from *Pseudomonas aeruginosa* (Kadurugamuwa and Beveridge 1995), *Porphyromonas gingivalis* (Haurat et al. 2011) and *Escherichia coli* (Lee et al. 2018). Alongside the detection of LPS, the phospholipid composition of OMVs has also been analysed. In those produced from *E. coli*, this is reported to be comparable to that of the OM with ∼80% phosphatidylethanolamine (PE), 15% phosphatidylglycerol (PG) and 5% cardiolipin (CL) also present (Horstman and Kuehn 2000; Horne et al. 2020). Compared to the phospholipid composition of whole *E. coli* cells (75% PE, 20% PG and 5% CL [Raetz and Dowhan 1990]), there is a slightly elevated proportion of PE.

OMV production also has physiological relevance for non‐pathogenic bacteria, with a role in nutrient acquisition (Roier et al. 2016), achieved by preferentially packaging certain enzymes into OMVs (Evans et al. 2012). Hypervesiculation can occur as a result of stress, particularly in the case of accumulation of peptidoglycan fragments in the periplasm (McBroom et al. 2006), LPS in the OM (Schwechheimer et al. 2014) or toxic/misfolded proteins (Schwechheimer and Kuehn 2013). Extensive studies have described knockout strains, which have a hypervesiculation phenotype, ultimately leading to the development of an *E. coli* strain, based on the BL21(DE3) strain used for recombinant protein production, which is able to produce up to three‐fold more OMVs. This strain, BL21(DE3)Δ60, has been shown to be capable of targeting recombinant proteins to the vesicles (Zanella et al. 2021).

The BL21(DE3)Δ60 strain reflects a broader trend in biotechnology of exploiting bacterial OMVs, particularly in vaccine development, due to their ability to elicit an immune response and their potential as targeted antigen delivery systems (e.g. Garling, Auvray, et al. 2025; Garling, Goursat, et al. 2025). For this, translocation of the recombinant protein into the periplasm is required, through the use of an appropriate signal peptide. Proteins that must fold in a reducing environment are transported via the Tat pathway, fully folded, whereas those which require disulfide bond formation, an oxidising process, are transported in an unfolded state through the Sec pathway (Dalbey and Kuhn 2012). This dependence on periplasmic disulfide bond formation, catalysed by the DsbA/DsbB system (Bardwell et al. 1991), which is targeted into the periplasm by an amino terminal signalling peptide (ssDsbA) (Dinh and Bernhardt 2011; Schierle et al. 2003), represents a fundamental limitation of recombinant protein production in *E. coli*.

This, and other limitations including issues with low solubility and yield, can be overcome by using the vesicle nucleating peptide (VNp) technology (Eastwood et al. 2023). This innovative method, developed from a serendipitous discovery made while studying amino‐terminal acetylated recombinant proteins in *E. coli*, uses a short peptide tag, derived from human synucleins, to target recombinant proteins into the lumen of membrane‐bound vesicles (Eastwood et al. 2023). These vesicles can be either extracellular or intracellular, with intact vesicles having been observed free‐floating in the cytoplasm, which is, to the best of our knowledge, the first observation of its kind in bacteria. VNp fusions significantly increase the yield of a protein of interest (POI), with several VNp variants having been produced to maximise POI yield. VNp2, VNp6 and VNp15 are currently the most commonly used due to their protein‐packaging efficiency (Streather et al. 2023). This technology also allows for the production of otherwise toxic or insoluble proteins, including disulfide bond‐containing antibodies (Baker et al. 2025), through compartmentalisation. The extracellular vesicles (EVs) provide a stable storage environment and simplify downstream processing with the POI being the most abundant protein contained within them. Bacterial cells can be easily removed from the culture by centrifugation, leaving vesicles in the supernatant and reducing the number of contaminants. Purified recombinant proteins from VNp vesicles are properly folded and functional (e.g. Eastwood et al. 2023; Baker et al. 2025; Baker and Mulvihill 2025; Mutailifu et al. 2025; Puhringer et al. 2026; Qing et al. 2024; Wu et al. 2024). Its many advantages make VNp attractive for numerous applications across biotechnology, as recombinant proteins can be simply released from the EV and purified away from EV‐associated endotoxin using well‐established methods (Aida and Pabst 1990; Liu et al. 1997; Magalhaes et al. 2007).

VNp has proven successful with a wide range of POIs but little is known about the biophysical properties of VNp induced vesicles (VNp‐EVs), their relationship to OMVs or their mechanism of formation. Currently, it is predicted that in the formation of EVs, VNp is propsed to interact with the bacterial membrane and promote outward membrane curvature, which continues until the membrane fuses back on itself, the vesicle buds off and is released into the media. It is unclear if it first interacts with the inner membrane (IM), causing subsequent bulging of both membranes and resulting in vesicles with two membrane layers or whether the VNp fusion is translocated to the periplasm prior to inducing outward curvature of the OM only, like OMVs. The determining factor for intracellular or EV formation is also unknown. The aim of this work is to deepen the understanding of VNp‐EV composition to give mechanistic insight and inform future uses of the technology. We also aimed to provide more evidence to support the hypothesis that expression of the VNp tag directs the protein to which it is fused into EVs rather than simply causing a hypervesiculation phenotype with release of random proteins.

We describe a detailed biophysical characterisation of VNp‐fusion induced vesicles and compare them with bacterial OMVs from cells expressing an ssDsbA fusion, henceforth differentiated as VNp‐EVs and ssDsbA‐OMVs. We find that while they share some similar lipid composition and both contain oxidising environments, there are several key differences. The luminal concentration of VNp‐fusion is significantly higher than that observed for OMVs containing mCherry fused to a periplasm targeting peptide. The higher luminal concentration in part explains why VNp‐EVs provide increased relative abundance and yield of the VNp‐ fusion compared to the ssDsbA fusion within OMVs, which not only simplifies downstream purification, but results in significantly higher yields of the target protein. These properties make them extremely attractive vehicles for the simple production and isolation of a wider range of recombinant protein than is normally allowed with the *E. coli* host system, and have the potential for a wide range of biotechnology applications. The results of this work also allowed for a revised mechanism of VNp‐EV formation to be hypothesised and therefore better inform its applications.

## 2 Materials and Methods

### 2.1 Bacterial Cell Culture and Protein Induction

BL21 Star (DE3) *E. coli* cells (F^−^ *omp*T *hsd*S_B_ (r_B_^–^, m_B_^–^) *gal dcm rne131* (DE3)) were used for all experiments. *E. coli* cells were cultured using LB (10 g Tryptone; 10 g NaCl; 5 g Yeast Extract [per litre]) and TB (12 g Tryptone; 24 g Yeast Extract; 4 mL 10% glycerol; 17 mM KH_2_PO_4_ 72 mM K_2_HPO_4_ [per litre]) media each supplemented with kanamycin (50 µg/ml final conc.). 5 mL LB starters from fresh bacterial transformations were cultured at 37°C to saturation and used to inoculate 25–500 mL volume TB media flask cultures that were incubated overnight at 30°C with 200 rpm orbital shaking (2.5 cm throw). Oxygenation of cultures was maximised, to promote vesicle production, by maintaining a large liquid surface area during growth (e.g. 25 mL media in 500 mL conical flask or 500 mL/1 L of media in a 5 L conical flask). Recombinant protein expression from the T7 promoter was induced by addition of IPTG to a maximum final concentration of 20 µg/mL (84 µM) once the culture had reached an OD_600_ of 0.8–1.0. It should be noted that use of higher IPTG concentrations results in cell lysis. Growth curves were generated from 96‐well plate cultures, prepared from late log‐phase cultures, diluted into fresh media to an OD_600_ of 0.1 at the start of the growth analysis experiment. Fluorescence signal from overnight plate cultures was obtained using a BMG Labtech Clariostar plus shaking UV & fluorescence plate reader. Plates were incubated at 37°C using double‐orbital shaking, and 589 nm absorbance values were taken every 15 min for the duration of the experiment. Fluorescence curves were generated from averages of four individual biological repeats.

### 2.2 Molecular Biology

All tags and gene sequences were codon optimised for expression in *E. coli*, and synthesised DNA sequences (*Thermofisher*) were cloned into pRSFDUET‐1 plasmid (*Novagen*) using appropriate restriction enzymes. Bacterial expression plasmids described in this study are summarised in Table S1. Plasmids and plasmid sequences from this laboratory are made available from Addgene upon publication (https://www.addgene.org/Dan_Mulvihill/). Note: Stable recombinant EV production requires use of plasmids with antibiotic selections that do not target the cell wall.

### 2.3 Recombinant Vesicle Isolation

To isolate crude preparations of VNp‐EVs and ssDsbA‐OMVs from cultures, *E. coli* cells were initially separated from cultures by centrifugation at 3000 × *g* for 30 min. The subsequent vesicle containing media was passed through a 0.45 µm polyethersulfone (PES) filter and either analysed directly, stored at 4°C or concentrated using a 20 mL 50 kDa cutoff PES centrifugal concentrator (Fisher Scientific) by centrifugation at 500 × *g* to reduce volume from 25 to 1 mL. These will be referred to as ‘vesicle preps’ throughout the paper.

### 2.4 Dynamic Light Scattering

Was undertaken using an Anton Paar Litesizer DLS 501 at 25°C. A series of 10 particle size measurements were undertaken upon 1 mL of 0.45 µm filtered purified vesicle preps in either TB or 1 x PBS, prior to centrifugal concentration. Data were analysed using Kalliope software (Anton Paar) and weighted by both number of particles and intensity of scattering from particles. Polydispersity (PDi) values were calculated from the mean polydispersity index percentage measured from the sample using the equation: (%/100)^2^ to give a value ≤1 where 1 represents a very diverse distribution of particle sizes.

### 2.5 Protein Concentration Determination

Fluorescence scans were used to determine concentration of mCherry2 fusion proteins in vesicle containing media and soluble protein extracts. Absorbance was measured at 589 nm using a Varian Cary 50 Bio UV‐Vis spectrophotometer, with measurements from an equivalent empty vector culture used for baseline correction. Concentration was determined using an extinction coefficient of 79,400 M^−1^cm^−1^.

### 2.6 Preparation and Analysis of Phospholipids

Equivalent cell mass samples were isolated by centrifugation from timepoints during overnight *E. coli* cultures, chilled on ice and then rapidly frozen. Vesicles were isolated and concentrated as described above. Lipids were isolated by adding 300 µL of methyl‐tert‐butyl‐ether (MTBE): MeOH (10:3, v/v) per 50 mg cell pellet, mixed rapidly by shaking and vortex, prior to incubation with continuous shaking at RT for 1 h. dH_2_O was added to a final volumetric ratio of 10:3:2.5 (MTBE: MeOH: dH_2_O), then vortexed and incubated with continuous shaking at RT for 1 h. Samples were subsequently centrifuged at 1000 × *g* for 10 min at 4°C, and the top layer was extracted using a glass Pasteur pipette. The final lipid‐containing solvent was evaporated using a rotary evaporator (*Rotavap*) or under a stream of nitrogen, and the resultant dry lipids were stored at −20°C until required. Prior to thin layer chromatography (TLC), lipid samples were resuspended in either 500 µL (cells extract) or 50–100 µL (vesicle extract) of chloroform. The TLC chamber was equilibrated for at least 45 min prior to running the TLC by filling to ∼1 cm deep with the solvent (65:25:8 v/v chloroform: MeOH: Acetic acid), inserting a piece of filter paper, just smaller than the internal height/width of the tank, prior to replacing the lid, and allowing the TLC chamber to equilibrate. TLC plates (*Merck, Darmstadt, Germany*) were cut to size, a pencil line drawn 1–1.5 cm from the bottom and labelled appropriately with samples 1 cm apart (first and last samples at least 0.5 cm from the edge of the plate). Initially, 10 µL of each sample was loaded before determining ‘equal loading’ through densitometry using Image J (Schindelin et al. 2012). The plate was placed level on the bottom of the TLC tank, with the solvent below the pencil line and the lid of the tank replaced. Plates were allowed to develop until the solvent front was ∼1 cm from the top of the plate, and subsequently allowed to dry fully outside the tank, before staining with aerosolised Molybdenum Blue solution (*Merck, MO, USA*). Glycoprotein staining of SDS‐PAGE gels (for detection of LPS) was carried out using Pro‐Q Emerald 300 (Hart et al. 2003), following the manufacturer's protocol. NMR analysis of phospholipids purified from VNp‐fusion induced vesicles was undertaken as described elsewhere (Medina‐Carmona et al. 2020).

### 2.7 Transmitted Electron Microscopy (TEM) Thin Section Analysis of Vesicles

Crude EV preparations were isolated and concentrated as described above. The vesicles (approximately 100 µL) were resuspended in 2 mL of 2.5% (w/v) glutaraldehyde in 100 mM sodium cacodylate buffer pH 7.2 (CAB) and fixed for 2 h at RT with gentle rotation (20 rpm). Vesicles were washed twice into 100 mM CAB by buffer exchange using a 50 kDa cutoff PES centrifugal concentrator (Fisher Scientific) by centrifugation at 500 × *g*. Vesicles were post‐fixed with 1% (w/v) osmium tetroxide in 100 mM CAB for 2 h and subsequently washed twice with dH2O, by buffer exchange. Vesicles were dehydrated by incubation in an ethanol gradient, 50% EtOH for 10 min, 70% EtOH overnight, and 90% EtOH for 10 min, followed by three 10 min washes in 100% dry EtOH. Samples were then washed twice with propylene oxide for 15 min. Vesicles were embedded by re‐suspension in 1 mL of a 1:1 mix of propylene oxide and Agar LV Resin and incubated for 30 min with rotation. The vesicles were resuspended in fresh resin and transferred to a 1‐mL BEEM embedding capsule, and samples were polymerised for 20 h at 60°C. Ultrathin sections were cut using a Leica EM UC7 ultramicrotome equipped with a diamond knife (DiATOME 45°). Sections (70 nm) were collected on uncoated 400‐mesh copper grids. Grids were stained by incubation in 4.5% (w/v) uranyl acetate in 1% (v/v) acetic acid for 45 min followed by washing in a stream of dH_2_O. Grids were then stained with Reynolds lead citrate for 7 min followed by washing in a stream of dH_2_O. Electron microscopy was performed using a JEOL‐1230 transmission electron microscope operated at an accelerating voltage of 80 kV equipped with a Gatan One View digital camera. Images from a minimum of four biological and technical repeats where thousands of vesicles were visualised, and true representative micrographs are shown here.

### 2.8 Cryogenic Electron Microscopy of VNp Induced Vesicles

Crude EV preparations were isolated and concentrated as described above. Quantifoil Lacey carbon 300 mesh Cu grids were glow‐discharged for 60 s at 15 mA in a PELCO easiGlow device. Vesicles were applied to the grid in a 4 uL sample and blotted with a Vitrobot Mark IV, using the following settings 20 blot force, 3 s blotting, 100% humidity at 4°C. Sample grids were imaged using a Thermofisher Glacios 200 kV microscope equipped with X‐FEG electron gun, Selectris energy filter with a 20 eV slit width, and Falcon 4i camera. Samples were imaged at 79,000x magnification at a dose of 15 *e* ^−^/Å^2^, resulting in images with 0.16 nm pixels.

### 2.9 Widefield Fluorescence Microscopy

Cells were mounted onto coverslips under <1 mm thick circular soft 1% agarose pads and attached with appropriate spacers onto glass slides (Mulvihill 2017), and the remaining gap was flooded (using capillary action) with TB supplemented with kanamycin and 20 µg/mL IPTG. Samples were subsequently visualised on an inverted widefield fluorescence imaging system (Baker et al. 2016). All live cell imaging was completed within 30 min of samples being mounted onto coverslips. RADA stain (R&D Systems (Biotechne), MN, USA) was added to samples at a final concentration of 125 µM and incubated for 1 h at 37°C before fixation with ethanol.

### 2.10 Structured Illumination Microscopy (SIM)

Structured Illumination Microscopy (SIM) was undertaken using a Zeiss Elyra PS 1 microscope with either a 100x NA 1.46 or 63x NA 1.4 oil immersion objective lens (Zeiss α Plan‐Apochromat) as described previously (Eastwood et al. 2023; Brooker et al. 2025). Cells and vesicles were mounted as described above, using high precision No.1.5 coverslips (*Zeiss, Jenna, Germany*). A 405 nm laser was used to illuminate FROG/B, while 488 and 561 nm lasers were used to illuminate mNeongreen and mCherry2 fusions, respectively. The optical filter set consisted of laser blocking filter MBS 405/488/561 as the dichroic mirror, and the dual‐band emission filter LBF‐488/561. A total of 3/5 rotations of the illumination pattern were implemented to obtain two‐dimensional information. During time lapse experiments, cells were maintained at 37°C with constant humidity using an INU incubation system (Tokai Hit, Shizuoka‐ken, Japan). Super‐resolution SIM image processing was performed using the Zeiss Zen software. Two‐colour images were aligned using the same software following a calibration using a pre‐mounted MultiSpec bead sample (*Zeiss, Jenna, Germany*). Pearson's correlation coefficient (*r*) of fluorescence signals was calculated from signal aligned SIM images using the Fiji software (Schindelin et al. 2012) coloc2 analysis tool with Costes threshold regression and 10 randomisations (Figure S1).

### 2.11 Fluorescence Lifetime Imaging Microscopy (FLIM)

The one‐ and two‐ photon systems used in this work have been previously described (Eastwood et al. 2023; Botchway et al. 2015). Prior to FLIM data acquisition, protein expression levels were verified using confocal microscopy. Here, a Nikon Eclipse C2‐Si confocal scan head attached to an inverted Nikon TE2000 or Ti‐E microscope (Nikon, Tokyo, Japan) was used. mNeongreen and mCherry2 fluorescent protein (FP) were excited at 491 nm (emission 520/35 nm) and 561 nm (emission 630/50 nm), respectively, using an NKT super continuum laser. For both one and two‐photon excitation, emission was collected by the same objective through filters (above) and detected with an external hybrid detector module (HPM‐100‐40, *Becker & Hickl, Germany*), that links a GaAsP photomultiplier tube (PMT) with a time correlated single photon counting (TCSPC) module (SPC‐QC‐104, *Becker and Hickl, Germany*). Photon counts of at least 1000 were used for the multi‐exponential analysis. Raw time correlated single photon counting decay curve at each pixel (256 × 256 or higher) of the images were analysed using SPCImage software v.8.8 (*Becker and Hickl, GmbH*); a single exponential fit model with a laser repetition time value of 25 ns was used for the decay curve fitting (Figure S2). *Vesicular concentrations* of mCherry fusions were determined as described previously (Bisby et al. 2012; Botchway et al. 2008) using a one photon excitation FLIM system, equipped with a SuperK EXTREME NKT‐SC 470–2000 nm supercontinuum laser (NKT Photonics), which generates at a variable repetition rate with 70 ps pulse width. The desired wavelengths were selected using a SuperK SELECT multi‐line tunable filter (NKT photonics). Images were collected through a 100 × 1.49 NA TIRFM oil objective (Nikon). Laser power was set at 20%, and images were captured using a large pinhole, 20 µm field zoom, 5 µs pixel dwell time, over a 512 × 512 pixel area. Image data was analysed with binning set at 3. A photon#—FP concentration calibration equation was generated from photon measurements averaged from five separate FLIM acquisitions of samples of known concentrations of FP dissolved in PBS. To ensure consistency between calibration solution measurements, data were captured 2 µm above the coverslip. Average photons/pixel gate was measured for ∼40 separate vesicles from three separate fields of view per sample. Larger objects (typically > 2 x signal from average), likely corresponding to vesicle aggregates, were omitted from the measurements. Average photon values were converted to FP concentration using the previously determined calibration equation. *Anisotropy* values were determined on the above system (using a 60 × 1.27 NA water objective lens), as described previously (Smith et al. 2024). This objective was chosen over the 100x NA 1.49 since the latter is known to introduce large errors for anisotropy measurements. Two‐photon excitation anisotropy was employed as the maximum value was 0.57 compared to 0.4 for one‐photon excitation. *Viscometry* was determined as described previously (Clancy et al. 2023) on the above multiphoton system using 910 nm excitation. A lifetime—viscosity calibration equation was calculated using equivalent mNeongreen samples dissolved in increasing w/v glycerol solutions (Figure S3).

## 3 Results

We have previously described a simple peptide tagging methodology by which the addition of a short VNp sequence to the amino terminal of a protein results in the vesicular export of the resultant recombinant fusion protein from *E. coli* (Eastwood et al. 2023). This system has been applied to a wide range of proteins, resulting in high‐yield production and simplified purification of diverse recombinant proteins (e.g. Baker et al. 2025; Mutailifu et al. 2025; Wu et al. 2024; Mekhtieva et al. 2024; Liu et al. 2025), yet their biophysical organisation and how these vesicles relate to the naturally occurring *E. coli* OMVs remains unknown. In an attempt to address this, we generated fusions between a bright monomeric fluorescent protein, mCherry2 (mCh2) (Shen et al. 2017), with either an amino terminal VNp6 tag (MDVFKKGFSIADEGVVGAVEKTDQGVTEAAEKTKEGVM) (Eastwood et al. 2023), or a native ssDsbA signalling peptide (MKKIWLALAGLVLAFSASAAQ) that directs co‐translational export of proteins through the signal recognition particle (SRP)‐dependent translocation pathway (Dinh and Bernhardt 2011; Schierle et al. 2003) into the periplasm from where it is subsequently incorporated into OMVs. VNp6 is a modified variant of human γ‐synuclein, designed to optimise export of VNp fusion proteins into VNp‐EVs. Expression from these VNp6‐mCh2 and ssDsbA‐mCh2 constructs resulted in the production of mCh2 filled vesicles (Figure 1a,b) within the media of the *E. coli* culture.

**FIGURE 1:**
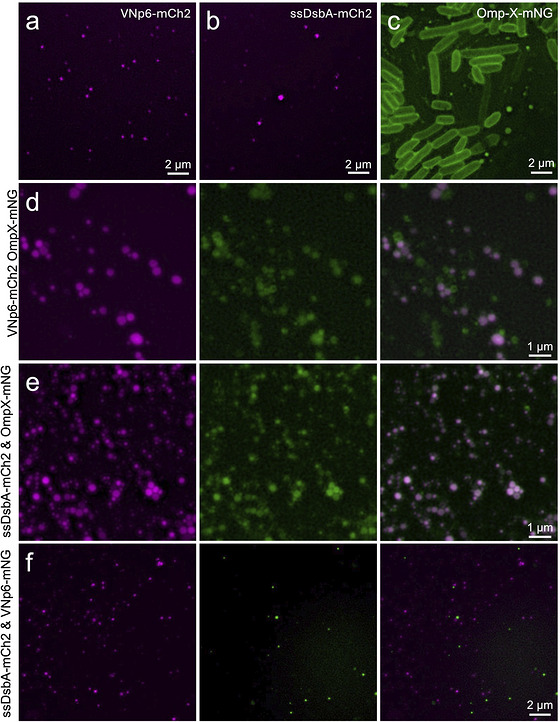
Structured Illumination Microscopy of *E. coli* vesicles. Vesicles isolated from BL21 DE3 cells expressing (a) VNp6‐mCh2, (b) ssDsbA‐mCh2. (c) A SIM image of a culture of cells expressing fluorescently tagged outer membrane protein, OmpX, highlights the OMV production from *E. coli*. Vesicles isolated from cultures of *E. coli* expressing (d) VNp6‐mCh2 and OmpX‐mNG, (e) ssDsbA‐mCh2 and OmpX‐mNG, (f) or ssDsbA‐mCh2 VNp6‐mNG.

### 3.1 VNp‐EVs Contain Luminal and Membrane Proteins Concurrently and Are Produced via a Distinct Mechanism From OMVs

To simultaneously follow vesicle content and outer membrane organisation in cells and vesicles, constructs were generated allowing co‐expression of the above proteins with the *E. coli* outer membrane protein OmpX fused to mNeongreen (mNG) (Shaner et al. 2013) at its carboxyl terminus. OmpX is an integral small beta‐barrel outer‐membrane protein (Vogt and Schulz 1999), with both amino and carboxyl termini ending in the periplasm. Expression of this construct revealed OmpX‐mNG localises to the membrane of bacterial cells and EVs within the culture media (Figure 1c). Dynamic light scattering (DLS) analysis was consistent with the size of vesicle particles in the media. SIM was used consistently in this work for its ability to visualise fluorescent protein tagged samples at sub‐diffraction‐limited resolution, particularly important for observing membrane organisation in bacterial cells and EVs. SIM timelapse analysis of cells expressing both VNp6‐mCh2 and OmpX‐mNG revealed mNG associated vesicles. The VNp‐EVs formed rapidly, typically within a minute, and concurrently filled with mCh2 protein (Figure S4).

Vesicles isolated from cells co‐expressing OmpX‐mNG with either VNp6‐mCh2 or ssDsbA‐mCh2 possessed OmpX‐mNG associated membranes (Figures 1d,e & S4), with the majority (>95%) of both VNp6‐mCh2 and ssDsbA‐mCh2 fusion foci seen located within OmpX‐mNG vesicles (Figures 1 & S1). While the majority of OmpX‐mNG vesicles contained ssDsbA‐mCh2 signal (Figures 1e & S1), less than half of OmpX vesicles contained VNp‐mCh2 in cultures co‐expressing both VNp‐mCh2 and OmpX‐mNG (Figures 1d & S1). This indicates the presence of two different population of vesicles, with ssDsbA‐mCh2 containing periplasm targeted to almost all the OmpX‐mNG OMVs, while recombinant VNp‐fusion localised to a subpopulation of vesicles, nucleated by the VNp in a mechanism distinct from that resulting in OMV biogenesis. To test this hypothesis, a construct was generated to allow simultaneous co‐expression of ssDsbA‐mCh2 (magenta) and VNp‐mNG (green) (Figure 1f). In all samples examined, the signals were never observed colocalised within the same vesicle (Figures 1f & S1). These results are consistent with the VNp‐ and ssDsbA‐ FP fusions recruiting to separate and distinct, vesicle populations.

Fluorescence lifetime imaging microscopy (FLIM) was employed to determine anisotropy values of fusion‐proteins within EV preparations from cultures of *E. coli* expressing VNp‐mNG ± OmpX, or ssDsbA‐mNG, in order to establish oligomerisation status and dynamics (Table 1). This technique measures the time a fluorophore remains in the excited state before releasing a photon and returning to the ground state (lifetime), which is affected by its local environment and is independent of its concentration, unlike standard microscopy intensity measurements. Consistent with previous immuno‐EM analysis (Eastwood et al. 2023) VNp‐fusions were soluble monomers within the vesicle lumen (anisotropy—0.312 mA ± 0.025) (Teijeiro‐Gonzalez et al. 2021), whereas vesicle‐associated OmpX‐mNG had a lower anisotropy (0.203 mA ± 0.04), consistent with integration within the vesicle membrane (Figure 1c). These anisotropy values are consistent with those previously determined for monomeric and dimeric fluorescently tagged proteins (Teijeiro‐Gonzalez et al. 2021). Surprisingly, ssDsbA fusions appear as dimers within the vesicle (anisotropy value: 0.229 mA ± 0.081).

**TABLE 1:** Properties of VNp induced vesicles and ssDsbA containing OMVs. Diameter and Polydispersity Index value were determined using DLS. Anisotropy, vesicular viscosity, and concentration of vesicular mCh2 were determined using FLIM. Percentage of total vesicular protein taken up by fusion protein determined by SDS‐PAGE densitometry of crude EV preparations. Concentration of mCh2 in the supernatant determined by spectroscopic absorbance scan and Beer‐Lambert law. Number of mCh2 molecules per vesicles, concentration of mCh2 and concentration of vesicles in culture supernatant calculated using other values in this table and the Avogadro constant.

Construct	VNp6‐fusion	ssDsbA‐fusion	VNp6‐fusion + OmpX
Diameter (nm)^a^	163 ± 2.5	144 ± 2.6	164 ± 1.9
Polydispersity Index value^a^	0.079	0.077	0.072
Anisotropy of vesicular fusion protein^b^	0.312 ± 0.025	0.229 ± 0.081	—
Vesicular Viscosity (centiPoise)^b^	7.677 ± 0.020	8.093 ± 0.011	6.489 ± 0.024
[Vesicular mCherry2] (µM)^c^	7.23 ± 0.83	5 ± 0.72	9.29 ± 1.304
% total vesicular protein^c^	76%	37%	85%
# mCherry2 molecules/vesicle^c^	7.69	3.54	10.1
mCherry2 in media (µM)^c^	9.14 ± 2.17	1.39 ± 0.14	13.26 ± 0.84
[Vesicles] (µM)^c^	1.19	0.39	1.32

^a^ Determined by dynamic light scattering.

^b^ Determined with mNeongreen fusions.

^c^ Determined with mCherry2 fusions.

### 3.2 VNp‐EVs and OMVs Both Originate From the *E. coli* Outer Membrane and Are Comparable in Size

To provide insight into the origin of the vesicle membranes, we examined the phospholipid composition of the VNp‐EVs and OMVs. Samples were taken at timepoints from *E. coli* cells expressing a VNp‐fusion protein and crude vesicle preparations taken from the subsequent culture media. Phospholipids were purified from the samples and analysed by TLC. Molybdenum blue staining of the chromatography plates revealed the presence of PE, PG and CL (Figure 2a), the relative abundance of which varied between samples (Figure 2b). As reported previously the relative abundance of CL within *E. coli* increased as the experiment progressed, making up 36% of the membrane lipid in the stationary phase cells (Hiraoka et al. 1993). In contrast, CL made up only 7% of the phospholipid composition of VNp induced vesicles. 83% of the vesicle membranes was made up of PE lipid, which is a significantly higher proportion than seen in *E. coli* cells in any growth phase (Figure 2a,b) (Horstman and Kuehn 2000; Horne et al. 2020; Raetz and Dowhan 1990). The relative PG composition of membranes did not vary dramatically between samples. NMR spectra of VNp vesicle phospholipid analysis and comparison with fingerprint spectra of phospholipid standards (Medina‐Carmona et al. 2020) confirmed the composition and relative abundance of phospholipids within the vesicle membrane (Figure 2d) were consistent with the TLC analysis. When phospholipid composition between VNp‐EVs, and OMVs were analysed, the relative lipid composition did not vary significantly between samples (Figure 2c), suggesting a similar cellular origin, and all were consistent with the previously reported composition of the *E. coli* OM (∼80% PE, ∼15% PG and ∼5% CL) (Horne et al. 2020).

**FIGURE 2:**
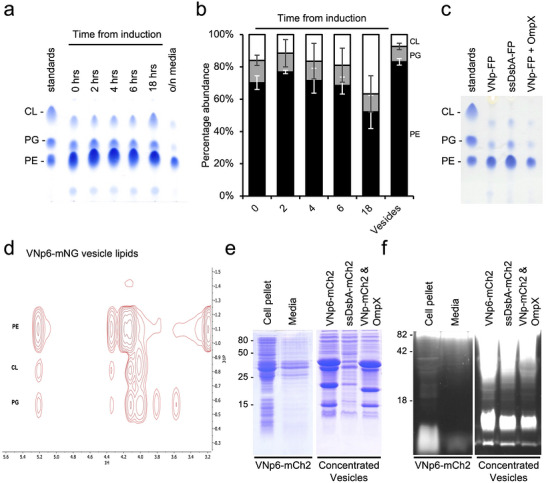
Lipid analysis of VNp‐fusion induced vesicles and ssDsbA‐OMVs. (a) Molybdenum blue stained TLC plate with phospholipid extracts from a time course of cells expressing VNp6‐mCh2 (time from IPTG induction), and vesicle lipid from the overnight media. (b) Quantification of relative PE (black), PG (grey) and CL (white) phospholipid levels, averaged from three biological repeats of experiment show in (a). (c) Molybdenum blue stained TLC plate with phospholipid extracts from vesicles isolated from cultures of *E. coli* expressing VNp6‐mCh2, ssDsbA‐mCh2 and VNp6‐mCh2 OmpX‐mNG. (d) 2d NMR spectra of phospholipid extracts from VNp6‐mCh2 induced vesicles. Coomassie blue (e) and Pro‐Q Emerald 300 (f) stained SDS‐PAGE gels of cell pellet and culture media from an overnight culture of *E. coli* expressing VNp6‐mCh2, or concentrated vesicles isolated from overnight cultures of bacteria expressing VNp6‐mCh2, ssDsbA‐mCh2 or VNp6‐mCh2 OmpX‐mNG.

As an additional analysis of the vesicle membranes, equivalent samples of *E. coli* cells, culture media and vesicle samples were subject to SDS‐PAGE. Duplicate gels were stained with either coomassie‐G250, to highlight protein composition (Figure 2e), or Pro‐Q Emerald 300 (Figure 2f), to confirm the presence of LPS and verify the *E. coli* outer‐membrane origin of the vesicle membranes. The VNp and ssDsbA fusion bands were present in each sample, but the relative abundance of the two fusions compared to endogenous *E. coli* proteins differed significantly (Figure 2e). In contrast, there was minimal variation in the relative abundance of LPS in each vesicle preparation (Figure 2f), again indicating the membranes from these different vesicles had a common cellular origin.

It is widely reported that the size of OMVs, as determined by EM and DLS, can vary from 20 to 250 nm in diameter (Guangzhang et al. 2023; Kolling and Matthews 1999; Ojima et al. 2024). DLS analysis of crude VNp‐EV and ssDsbA‐OMV preparations revealed (a) Polydispersity Index values of less than 0.1, indicating the samples were monodisperse, and (b) the VNp‐mCh2 vesicles were significantly larger than the ssDsbA‐OMVs (Table 1). Interestingly, co‐expression of VNp‐mCh2 and OmpX‐mNG resulted in no significant difference in vesicle size (163 ± 2.5 nm vs 164 ± 1.9 nm).

We went on to examine the membrane organisation and vesicle size distribution of the vesicles using both TEM and cryo‐EM imaging (Figure 3). Both TEM images of negatively stained thin sections (Figure 3a,b) and Cryo‐EM images of plunge frozen vesicles (Figure 3c) revealed overall membrane organisation was similar between samples, each containing a single lipid bilayer, with a thickness of approximately 5 nm across the constructs examined (Figure 5a), which is consistent with previously determined and published thickness of the *E. coli* outer membrane (Matias et al. 2003). Interestingly, as has been observed previously (Eastwood et al. 2023) but quantified here, a significant proportion of vesicles (∼5% overall) contained multiple membrane layers (Figure 3), which may be a reflection of vesicle formation, or due to an artefact of sample preparation. However, these data indicate that like OMVs, VNp‐EVs are encapsulated by membranes originating from the outer *E. coli* membrane.

**FIGURE 3:**
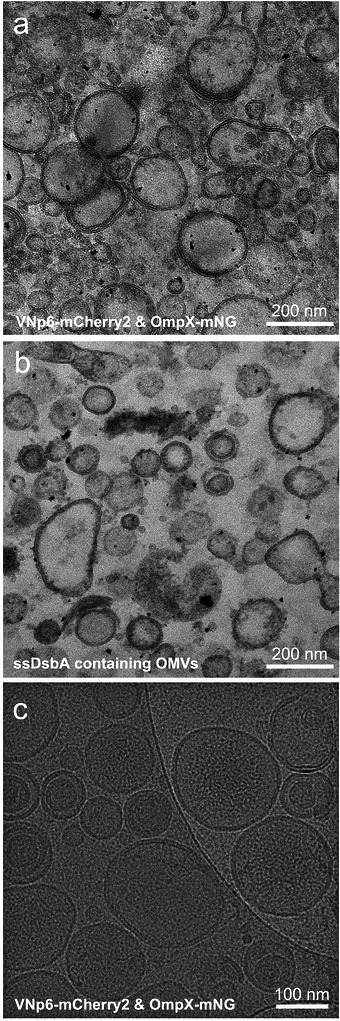
Negative stained thin‐section EM analysis of *E. coli* derived vesicles. EM images of negative stained thin sections of vesicles isolated from *E. coli* expressing (a) VNp6‐mCh2 and OmpX‐mNG or (b) ssDsbA‐mCh2. (c) Cryo‐EM image of vesicles isolated from *E. coli* expressing VNp6‐mCh2 and OmpX‐mNG.

To confirm which subcellular region the lumen of VNp‐EVs originated from, we employed the synthetic biosensor FROG/B (Sugiura et al. 2020) to characterise the redox environment within the vesicle lumen. This biosensor has been engineered to emit fluorescence at two wavelengths. While signal in the blue wavelengths is independent of the redox environment and therefore acts as a measure of local protein abundance, intensity in the green increases under oxidising conditions. Vesicles generated from cells expressing VNp‐FROG/B contain lumen with a more oxidised environment than the bacterial cytosol (Figure 4a,b). These data are consistent with the presence of disulphide bond containing VNp fusion proteins within both external and internal vesicles (e.g. human growth hormone, Etanercept, monoclonal antibodies, etc; Eastwood et al. 2023; Baker et al. 2025). Thus, the measured redox environment within the VNp induced vesicular lumen is consistent with an *E. coli* periplasmic origin. Interestingly, while rhodamine 3‐amino‐d‐alanine (RADA) staining, a tetramethylrhodamine‐based fluorescent D‐amino acid peptidoglycan dye, highlighted the periplasm of *E. coli* cells, no detectable RADA signal was observed in VNp‐mNG vesicle preparations (Figure 4c,d), suggesting peptidoglycan is absent from the vesicles, which is consistent with our previous mass‐spectrometry based analysis (Eastwood et al. 2023).

**FIGURE 4:**
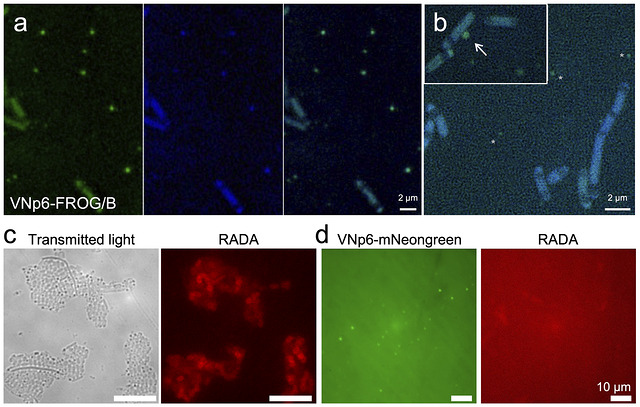
The environment within VNp induced extracellular vesicles. Widefield fluorescence (a) and Structured Illumination Microscopy (b) images of overnight cultures of *E. coli* cells expressing a fusion between VNp6 and the REDOX potential biosensor, FROG/B. These data illustrate the luminal environment is more oxidising (more green fluorescence) than the cytosol of the bacterial cells. Asterisks and arrows highlight vesicles in the SIM images. (c) While wide‐field RADA staining (red) highlights the periplasmic localisation of peptidoglycan within *E. coli* cells, (d) the dye is not detected in isolated VNp‐mNG (green) induced vesicles (Scales in ‘c’: 10 µm).

### 3.3 Vesicular Target Protein Yield Is Significantly Higher for VNp Fusions than ssDsbA Fusions

We next compared yields of recombinant proteins produced within VNp‐EVs and ssDsbA‐OMVs. We first measured the total fluorescence in cultures expressing these cargoes with and without OmpX‐mNG to control for potential differences in viability and stability (Figure S5). The final cell density of each of the cultures after 24 h was equivalent (not shown), indicating none of the constructs negatively impacted viability or growth of cells. In contrast, cultures containing cells expressing VNp6‐mCh2 had a maximal fluorescence at 589 nm six times higher than those expressing ssDsbA‐mCh2. Interestingly, while co‐expression with OmpX‐mNG had no significant impact on fluorescence dynamics in cells expressing ssDsbA‐mCh2, co‐expression with VNp6‐mCh2 resulted in a higher overall maximal fluorescence, that remained stable after the culture reached stationary phase (Figure S5).

We then determined the quantity of the target protein in each culture that was exported into the media by measuring the absorbance at the maximal extinction wavelength for the fluorescent proteins, mCh2 (589 nm) and mNG (506 nm) of media in which cells had been removed by centrifugation (Figure 5b). Average concentrations of mCh2 fusion‐proteins within vesicles in the culture media were subsequently calculated from a minimum of three independent biological repeats (Figure 5c). The VNp‐fusion resulted in 298 ± 70 mg/L yields of vesicle encapsulated mCh2 protein exported into the media, while the periplasmic targeting ssDsbA‐fusion resulted in 42 ± 4 mg/L yields. These data show that the VNp tag not only resulted in a higher yield of protein in the total culture (Figure S5), but a significantly higher proportion of the resultant fusion protein was exported into vesicles compared to equivalent protein targeted to the *E. coli* periplasm. Interestingly, co‐expression of OmpX‐mNG (or untagged OmpX—not shown) with either VNp or ssDsbA fusions, resulted in a reproducible and significant increase in the extracellular yield of mCh2 (Figure 5c).

**FIGURE 5:**
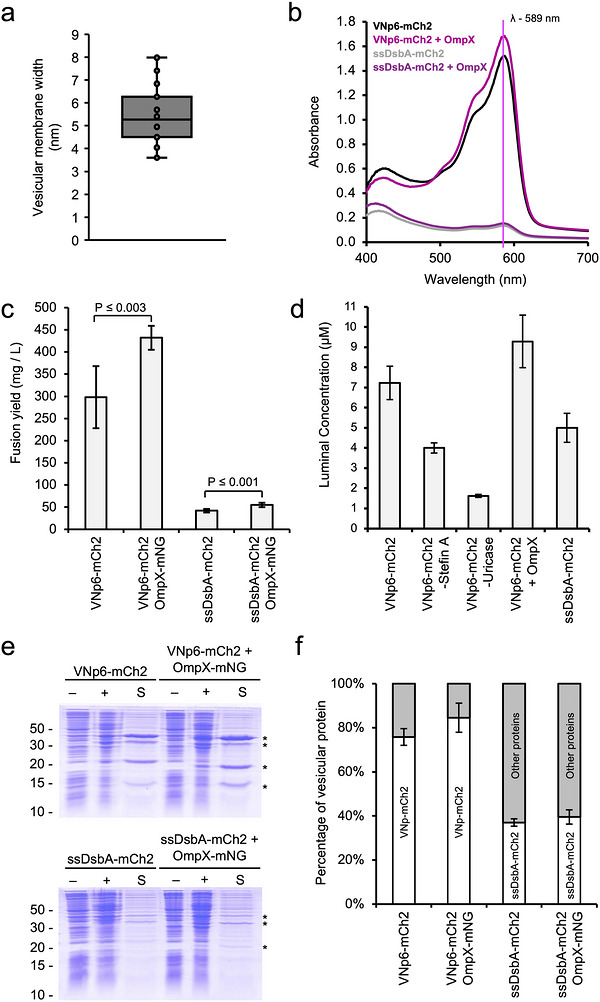
Characterisation of size and protein content of VNp vesicles and OMVs from *E. coli*. (a) Thickness of membrane of VNp6‐mCh2 induced vesicles, determined from TEM thin section and Cryo EM data. (b) Typical absorbance spectra of media from cultures of cells expressing VNp6‐Ch2 (black), VNp6‐mCh2 & OmpX (pink), ssDsbA‐mCh2 (grey) or ssDsbA & OmpX (purple). (c) Average yields calculated from spectra shown in (b), calculated from >3 biological repeats. (d) Luminal concentration of mCh2 signal within vesicles isolated from cultures of *E. coli* expressing VNp6‐mCh2, ssDsbA‐mCh2, VNp6‐mCh2 OmpX, VNp6‐mCh2‐StefinA or VNp6‐mCh2‐uricase, as determine by FLIM analysis. (e) Coomassie stained SDS‐PAGE gels showing preinduction (−), overnight post‐induction (+) cell pellet, and overnight supernatant (S) samples from cultures of *E. coli* expressing either VNp6‐mCh2, ssDsbA‐mCh2, VNp6‐mCh2 OmpX‐mNG, or ssDsbA‐mCh2 OmpX‐mNG. *—highlights bands that correlate with western blots of control mCh2 samples (Figure S5c). (f) Fraction (%) of total vesicular protein that is mCh2 (white) compared to other proteins (grey), averaged from three biological repeats of experiment show in (e).

### 3.4 The Luminal Concentration of VNp Fusion Proteins Is Higher than ssDsbA Fusion Proteins

FLIM was, again, employed to determine whether these differences in protein yield were a result of differences between constructs in the quantities of fusion protein packaged into vesicles, or the number of vesicles produced. FLIM provides a robust method for determining concentrations of fluorescently tagged proteins within a sample (Bisby et al. 2012; Botchway et al. 2008), taking into account the environment of the protein, such as protein‐protein interactions and quenching. Steady‐state imaging alone does not accurately give this information. Simply, a highly viscous environment (e.g. due to a high protein concentration) leads to a reduction in the emission lifetime of fluorescent proteins. This method is not dependent on size of the object, as the concentration is calculated from the number of photons gathered at the defined pixel gate, and thus removes bias brought about by sample aggregation. Vesicular concentrations of VNp‐fusions were determined using a previously described one photon excitation FLIM system (Eastwood et al. 2023; Botchway et al. 2015). Average photons/pixel gate was measured for ∼40 separate vesicles from three separate fields of view per sample, and average photon values were converted to fusion protein concentration using a calibration equation calculated using samples containing known concentrations of purified mCh2 protein. Using this method, we determined the luminal concentration for each fusion protein (Figure 5d, Table 1). Interestingly, these values followed the same trends observed in yields (Figure 5c,d) increasing from ssDsbA (5 ± 0.72 µM), to VNp6 (7.2 ± 0.83 µM), to VNp6 & OmpX (9.3 ± 1.3 µM).

At the same time, we examined the luminal concentration of VNp‐mCh2‐StefinA (43.4 kDa) and VNp‐mCh2‐Uricase (66.8 kDa) fusion proteins, compared to VNp‐mCh2 (30.9 kDa), to determine whether size of the fusion protein affected yield and luminal concentration of the VNp fusion. Stefin‐A and Uricase were selected as both have previously been produced and exported at high yield with the VNp system (Eastwood et al. 2023; Baker and Mulvihill 2025). A negative correlation was observed between the size of the fusion protein and both the luminal concentration (Figure 5d) as well as with the overall yield of each fusion protein from the culture (Figure S5b).

In addition to establishing luminal concentration of the fusion proteins, we also used FLIM to determine viscosity within the different vesicles. Using a recently established technique (Clancy et al. 2023), we examined the viscosity within vesicles generated from *E. coli* cells expressing either VNp6‐mNG, ssDsbA‐mNG, or co‐expressing VNp6‐mNG and OmpX (Table 1). mNG fusions were used, as this fluorescent protein is more sensitive to changes in viscosity than mCh2. Viscosity was the lowest in vesicles containing ssDsbA fusions (8.093 ± 0.011 centiPoise [cP]), increasing to 7.677 ± 0.020 cP for the VNp fusion (cP value increases inversely to viscosity), and was seen to be highest in vesicles containing VNp6‐mNG and OmpX (5 cP). While it is possible that using mNG rather mCh2 fluorescent proteins could bring about differences in yield, target enrichment and physical characteristics, it is safe to conclude viscometry increases in relation to the luminal concentration of the fusion protein.

The higher viscosity is consistent with the VNp fusion making up a significantly higher proportion of the overall vesicular content than the ssDsbA‐OMVs. To assess this further, we examined overall protein content in vesicles containing either VNp or ssDsbA mCherry2 fusions, to determine differences in the relative abundance of each fusion within them. As a control, the identity of Coomassie stained mCh2 and mNG protein bands were confirmed by western blot analysis (Figure S5c). We reproducibly observed that VNp‐fusions represent, on average, 80% of the total vesicle protein content (Figure 5e,f). In contrast, the ssDsbA‐fusion represented a significantly lower proportion of the overall protein content of OMVs, which contained an abundance of endogenous *E. coli* proteins (Figure 5e). This data are consistent with existing models where natural blebbing is brought about due to an increase in overall periplasmic content, rather than an induced nucleation and targeted vesicular delivery, observed with VNp‐EVs.

## 4 Discussion

In this study, we have compared recombinant vesicle nucleating peptide (VNp) induced vesicles (VNp‐EVs) (Eastwood et al. 2023) with OMVs containing a periplasmic targeted fusion protein, both produced from *E. coli*. The data indicate they are produced by distinct mechanisms, as VNp‐fusions are absent from ssDsbA‐OMVs, while ssDsbA‐fusion protein is not detected in VNp‐EVs, when co‐expressed (ssDsbA‐mCh2 and VNp6‐mNG). This exclusive segregation of molecules illustrates distinct mechanisms and is consistent with the observed differences in abundance and purity of the targeted fusion protein within the vesicles. Furthermore, the growth data showed that VNp fusion expression continues for a much longer period of time than ssDsbA fusion expression and reaches a higher maximal fluorescence (Figure S5). Moreover, the vesicular concentration of VNp fusions is significantly higher when compared to that of ssDsbA fusions (Figure 5d, Table 1). This implies that VNp is much more effective at targeting proteins of interest into EVs, produced a higher overall yield and strengthens the argument that they are produced via a different mechanism. Analysis of CryoEM and TEM images of VNp‐mCh2 vesicle preparations reveals these particles, are encapsulated within a single lipid bilayer. While VNp‐EVs were occasionally observed with multiple membrane layers (in less than 1 in 20 vesicles) and therefore represents a small subset of vesicles containing both inner and outer membranes, potentially brought about as an artefact of EM sample preparation. The presence of lipid‐A and relative proportion of PG and CL (Figure 2), as well as the thickness of the lipid bilayer, strongly indicate that as for OMVs, the VNp vesicle lipids originate from the outer *E. coli* membrane. This is consistent with the presence of outer membrane proteins within VNp induced vesicle preparations, but no IM proteins detected(25).

While OMV biogenesis is still not fully understood, genetic studies focussing on gene deletion strains have provided models to explain their formation. Strains lacking Lpp‐peptidoglycan cross‐links reduce vesiculation (Schwechheimer et al. 2014), whereas deletion of the outer membrane protein, OmpA, leads to increased OMV production. These effects are likely due to disruption between the OM and peptidoglycan (Deatherage et al. 2009; Sonntag et al. 1978). It is probable that OMV biogenesis requires a localised breakdown or disruption of the OM‐peptidoglycan cross‐links, otherwise it could only occur at regions where there are fewer crosslinks (i.e. at the cell poles). While OMV protein content can vary between strains and growth conditions, they typically contain periplasmic proteins, OM proteins and some molecular chaperones (Hong et al. 2019). Previously undertaken proteomic analysis of VNp‐EVs showed that the VNp fusion was the most abundant protein followed by OM proteins and some of the most highly abundant cytoplasmic proteins, such as DnaK, FusA and Mdh (Eastwood et al. 2023). The presence of OM proteins (OmpA/F), along with a lack of IM proteins and the presence of lipid‐A provide compelling evidence that these vesicles form from the outer membrane. Full length recombinant Synucleins (from which the VNp tags are derived) has been reported to localise to the periplasm through the SRP‐dependent pathway (Ren et al. 2007) when expressed recombinantly in *E. coli*, with the carboxy‐terminus being involved in periplasmic targeting. However, VNp tags are derived from the amino terminus of this protein. While no definitive mechanism for the translocation has been determined, with Sec and Tat pathways being the predominant means by which proteins are transported to the periplasm (Dalbey and Kuhn 2012), neither have been implicated here. One model is that once the VNp fusion is translocated into the periplasm it must pass through the peptidoglycan by diffusion to reach the outer membrane. It has been postulated that globular proteins of up to 50 kDa are able to readily pass through pores in the stretched *E. coli* peptidoglycan layer (Demchick and Koch 1996), where they would be able to associate with the outer membrane and induce formation and incorporation into EVs. This is consistent with our earlier published descriptions of the VNp system, that show larger proteins, and protein complexes tend to be incorporated into intracellular vesicles (Eastwood et al. 2023; Baker et al. 2025) (i.e. fail to pass across the peptidoglycan layer).

We postulate that, if the VNp peptide fusion is only able to access the IM (i.e. is unable to pass the peptidoglycan layer), it induces curvature of this membrane into the cytoplasm rather than curvature of the OM into the media. This explains why sometimes both intracellular and EVs are formed and that disulfide bond‐containing proteins (e.g. VNp‐mNG‐Etanercept [84 kDa]) have been observed in both vesicle types, showing that VNp fusions must be transported to the periplasm for this bond formation to occur. It is likely that molecular weight alone is an overly simplistic explanation for determining whether a VNp fusion will be exported or not. Other factors will have an impact, such as secondary/tertiary protein structure, overall shape and solubility. Consistent with this model, peptidoglycan would be absent from within the vesicles, as observed here (Figure 4d). We acknowledge the current limitations in techniques to establish the mechanism of VNp vesicle formation. Nevertheless, using methods including STED (*STimulated Emission Depletion*), STORM (*STochastic Optical Reconstruction Microscopy*), or MINIFLUX (*MINimal Fluorescence Photon Fluxes*), as well as future technologies as they evolve, it should be possible to gain higher‐resolution insight into this process.

While SIM timelapse imaging (Figure S4) does not provide the temporal and spatial resolution required to allow the capture of detailed membrane remodelling, it provides intriguing insights into the biomechanics of EV formation. To our knowledge, this represents the first super‐resolution timelapse description of vesicle formation from *E. coli* cells. Vesicles formed predominantly at the cell tips and site of cell division (Figure S4). Not only do the lipid composition, curvature, and dynamics of the membrane at these regions differ significantly from tubular regions of the cell, but the periplasm is also wider at these locations (Matias et al. 2003), allowing localised build‐up of the VNp‐fusion. Each of these factors are likely to be key in facilitating the rapid membrane reorganisation. Optimising conditions for the capture of vesicle formation highlighted key factors, other than minimising the impact of phototoxicity (Icha et al. 2017; Laissue et al. 2017), required to promote vesicle formation. These include maximising cell health prior to and post induction; inducing at the optimal cell density (i.e. OD_600_ 0.8–1.0); as well as maintaining temperature, hydration, and maximising oxygenation throughout the experiment, key factors for maximising vesiculation in flask cultures. While the VNp fusion has some effect upon vesicle formation kinetics, with optimised conditions we observed the rate of vesicle production increases significantly 5 h post‐induction. The size of some vesicles observed using SIM timelapse imaging was considerably larger than that measured using DLS and EM imaging of vesicle preparations. This is likely to be attributed to the fact that the cells cultured on an agarose pad are grown in a stable environment, allowing larger vesicle formation. In comparison, all vesicle preparations were taken from cultures of cells grown in shaking suspension, where earlier scission is promoted by the significantly higher shear forces within the liquid media, resulting in smaller, homogeneous vesicles (see Figure 1).

We show that the small beta‐barrel outer membrane protein, OmpX, localises to the surface of both VNp‐EVs and ssDsbA‐OMVs, consistent with the lipid bilayer originating from the bacterial outer membrane. Expression of additional OmpX or an OmpX‐mNG fusion increases both VNp‐fusion protein concentration within the vesicle as well as the overall yield within the culture. Our data suggests the additional OmpX does not impact the relative purity (Figure 5) of recombinant fusions within the VNp‐EVs or ssDsbA‐OMVs. Over‐expression of OmpX does not bring about the formation of significantly larger vesicles (Table 1). Therefore, it is likely to be due to a combination of more efficient packaging within each vesicle, as well as overall increase in membrane stability. The high concentration of fusion protein in VNp‐EVs compared to that in OMVs and the lack of VNp fusion present in ssDsbA‐OMVs when co‐expressed highlights the advantage of this technology to selectively target proteins into vesicles with significantly higher yield than other OMV engineering strategies. It also shows that it is not simply the case that VNp fusion‐expressing cells exhibit a hypervesiculation phenotype but rather the VNp fusion is driving vesicle formation. However, it is worth noting that expression of the ssDsbA‐fusion protein may lead to hypervesiculation, and changes to the biophysical properties of the OMV, compared to those produced from cells lacking the fusion.

In conclusion, the VNp‐EVs represent a distinct class of recombinant EVs from OMVs, that offer a simple and efficient route for producing high yields of relatively pure, concentrated, functional vesicle packaged recombinant protein, that can be applied to a wide range of proteins for industrial enzyme production and biotechnological applications. In addition,, due to the fact that VNp vesicles have been confirmed to contain lipid A, there is an exciting potential for their use in vaccine delivery.

## Author Contributions

B.R.S., T.A.E. and K.B. performed the experimental studies. M.L. performed TEM sample fixation, sectioning and imaging. A.E.B. performed FLIM imaging. T.T.v.d.V. performed Cryo‐EM sample fixation and imaging. L.J.C.J. supervised Cryo‐EM analysis. S.W.B. undertook FLIM data analysis. L.W. supervised SIM microscopy. D.P.M. supervised research, sought funding and managed the overall project. All authors designed experiments. D.P.M. wrote main drafts of the manuscript, and all authors contributed to editing.

## Conflicts of Interest

The authors declare no conflicts of interest.

## Supporting information




**Supporting Information**: jev270354‐sup‐0001‐SuppMat.docx

## Data Availability

The data that support the findings of this study are available on request from the corresponding author. The data are not publicly available due to privacy or ethical restrictions.
