# Supplementary material for: Vesicle Nucleation Peptide Fusion Induced Extracellular Vesicles Are Distinct From Escherichia coli Outer Membrane Vesicles, and Provide an Enhanced Platform for Protein Production and Purification
Source: J Extracell Vesicles. 2026 Aug 14;15(8):e70354. doi: 10.1002/jev2.70354 (PMC13475848; doi:10.1002/jev2.70354)
Supplement: Supplementary file 1 [file JEV2-15-e70354-s001.docx]

**Supplementary Data**

**Supplementary Figure 1.** **Colocalisation correlation analyses**. Example colocalization correlation analysis graphs between mNG and mCh2 signals from Structure Illumination Microscopy images of vesicles isolated from BL21 DE3 *E. coli* expressing (a) VNp6-mCh2 and OmpX-mNG, (b) ssDsbA-mCh2 and OmpX-mNG, or (c) ssDsbA-mCh2 and VNp6-mNG, as shown in Figure 1. Perfect colocalization between the green (y-axes) and red (x-axes) channels would present as a distribution that follows a line of equality, with a calculated Pearson correlation value (r) of 1.

**Supplementary Figure 2.** **Example calculated lifetime of FLIM acquisition of vesicles isolated from *E. coli* BL21 DE3 cells expressing VNp6-mNG.** (a) Analysed colour-coded FLIM map of VNp6-mNG containing vesicles. (b) Lifetime distribution histogram of total image from A. (c) Single exponential decay fit (pre-exponential factor a-100%), using 3x binning, of a pixel within highlighted circle within (a). Calculated lifetime (τ) of selected pixel is 2,247 ps. $\chi^{2}$ value of 1.25 indicative of a perfect single decay fit (black) to the data (red).

**Supplementary Figure 3. Calibration of viscosity dependent changes in mNG fluorescence lifetime**. An mNG concentration calibration equation generated from photon measurements averaged from 5 separate FLIM acquisitions of equivalent samples of purified fluorescent dissolved in different water: glycerol ratio solutions.

**Supplementary Figure 4. Frames from Structured Illumination Microscopy Timelapse videos of VNp-vesicle formation.** 1 minute time frames from Structured Illumination Microscopy timelapse videos of live *E. coli* co-expressing VNp6-mCh2 (magenta) and the outer membrane marker OmpX-mNG (green), from 6 individual cells (a – f). Scale bars: 1 µm.

**Supplementary Figure 5. Yield of vesicular packaged recombinant fluorescent protein tagged fusions.** (a) Fluorescence from overnight cultures of BL21 DE3 cells expressing VNp6-mCh2 (black circles), ssDsbA-mCh2 (grey crosses), VNp6-mCh2 OmpX-mNG (magenta circles), or ssDsbA-mCh2 OmpX-mNG (magenta crosses). Averages values as presented were calculated from 4 separate biological repeats. (b) Average yields of exported VNp-mCh2, VNp-mCh2-StefinA and VNp-mCh2-Uricase, each calculated from > 3 independent biological repeats. (c) Coomassie stained SDS-PAGE (left) and western blot (right) analysis of Ni^2+^ affinity purified mNG and mCh2 proteins. Commercial polyclonal anti-mNG and mCh2 primary antibodies (see materials and methods) were used.

**Supplementary Table 1. Summary of constructs used in this study.**

**Supplementary Table 1. Summary of expression constructs used in this study.**

| **Vector** | **pRSF-Duet**  **MCS 1** | **pRSF-Duet MCS 2** | **Expected phenotype** | **VNp-EVs?** | **OMVs?** | **Use** |
| --- | --- | --- | --- | --- | --- | --- |
| v1265 | VNp6-mCherry2 | – | mCh2 labelled cytoplasm and EV lumen | • | • | SIM, DLS, protein conc., lipid comp. |
| v1640 | VNp6-mCherry2 | OmpX-mNG | mCh2 labelled cytoplasm and EV lumen.  mNG labelled cell OM and OMV membrane | • | • | SIM, DLS, protein conc., lipid comp. |
| v1596 | ssDsbA-mCherry2 | – | mCh2 labelled periplasm and OMV lumen. |  | • | SIM, DLS, protein conc., lipid composition |
| v1650 | ssDsbA-mCherry2 | OmpX-mNG | mCh2 labelled periplasm and OMV lumen.  mNG labelled cell OM and OMV membrane |  | • | SIM, DLS, protein conc. |
| v1800 | – | OmpX-mNG | mNG labelled cell OM and OMV membrane |  | • | Widefield |
| v1670 | ssDsbA-mCherry2 | VNp6-mNG | VNp-EVs with mNG labelled lumen and OMVs. mCh2 labelled periplasm and OMV lumen | • | • | SIM |
| v1746 | VNp6-mCherry2-StefinA | – | mCh2 labelled cytoplasm and EV lumen | • | • | SIM, DLS, protein conc. |
| v1745 | VNp6-mCherry2-Uricase | – | mCh2 labelled cytoplasm and EV lumen | • | • | SIM, DLS, protein conc. |
| v1136 | VNp6-mNG | – | mNG labelled cytoplasm and EV lumen | • | • | Anisotropy, lipid comp. |
| v1822 | VNp6-mNG | OmpX | mNG labelled cytoplasm and EV lumen. OmpX overexpression | • | • | Anisotropy |
| v1595 | ssDsbA-mNG | – | mNG labelled periplasm and OMV lumen |  | • | Anisotropy |
| v1437 | VNp6-FROG/B | – | FROG/B labelled cytoplasm and EV lumen | • | • | Oxidation state analysis |
